# Assessment of Nutritional and Quality Properties of Leaves and Musts in Three Local Spanish Grapevine Varieties Undergoing Controlled Climate Change Scenarios

**DOI:** 10.3390/plants10061198

**Published:** 2021-06-11

**Authors:** Nieves Goicoechea, Leyre Jiménez, Eduardo Prieto, Yolanda Gogorcena, Inmaculada Pascual, Juan José Irigoyen, María Carmen Antolín

**Affiliations:** 1 Plant Stress Physiology Group, Department of Environmental Biology, School of Sciences, Universidad de Navarra, Associated to CSIC (EEAD, Zaragoza, ICVV, Logroño), 31008 Pamplona, Spain; ljimenez.12@alumni.unav.es (L.J.); eprieto.3@alumni.unav.es (E.P.); ipascual@unav.es (I.P.); jirigo@unav.es (J.J.I.); cantolin@unav.es (M.C.A.); 2 Departamento de Pomología, Estación Experimental de Aula Dei, Consejo Superior de Investigaciones Científicas (CSIC), 50080 Zaragoza, Spain; aoiz@eead.csic.es

**Keywords:** autochthonous grapevine varieties, elevated CO_2_, nutritional properties, phenolic compounds, primary metabolites, warming

## Abstract

The market demand together with the need for alternatives to withstand climate change led to the recovery of autochthonous grapevine varieties. Under climate change, the summer pruning of vineyards may lead to an increase of vegetative residuals of nutritional and medicinal interest. The objectives of our study were (1) to evaluate the nutritional properties of the leaves of three local Spanish grapevines (Tinto Velasco, TV, Pasera, PAS, and Ambrosina, AMB) when grown under climate change conditions, and (2) to test the potentiality of these grapevines as suitable candidates to be cultivated under climate change scenarios based on the quality of their must. Experimental assays were performed with fruit-bearing cuttings grown in temperature gradient greenhouses that simulate rising CO_2_ (700 μmol mol^−^^1^) and warming (ambient temperature +4 °C), either acting alone or in combination. TV and AMB were the most and the least affected by air temperature and CO_2_ concentration, respectively. The interaction of elevated CO_2_ with high temperature induced the accumulation of proteins and phenolic compounds in leaves of TV, thus enhancing their nutritional properties. In PAS, the negative effect of high temperature on protein contents was compensated for by elevated CO_2_. Warming was the most threatening scenario for maintaining the must quality in the three varieties, but elevated CO_2_ exerted a beneficial effect when acting alone and compensated for the negative effects of high temperatures. While TV may be a candidate to be cultivated in not very warm areas (higher altitudes or colder latitudes), PAS behaved as the most stable genotype under different environmental scenarios, making it the most versatile candidate for cultivation in areas affected by climate change.

## 1. Introduction

The search for highly productive vine varieties led to a varietal homogeneity in the vineyards, with the consequent loss of biodiversity. This high genetic uniformity puts in risk the production and the quality of grapes and wines in the context of climate change. During the last century, the air temperature in Spain has experienced an increase of 1.8 °C [1], which is over the predictions of global warming (1.5 °C) by 2030–2052 if it continues to increase at the current rate [2]. The market demand for regional diversity together with the need of searching alternatives to withstand climate change scenarios enhanced the interest for identifying and recovering forgotten autochthonous grapevine varieties in different Spanish regions [3,4,5,6,7]. The identification and exploitation of multistress-tolerant germplasm may be crucial to maintaining the viticulture in the Mediterranean areas under future climatic conditions [8].

Tinto Velasco (TV), Pasera (PAS), and Ambrosina (AMB) are local red grapevine (*Vitis vinifera* L.) varieties recovered in old vineyards (older than 65 years) in the north of Spain. Tinto Velasco produces medium bodied and pleasant wines, and Ambrosina, also known as Aubum in France, produces very fruity and fresh red wines. The oenological properties of the wines obtained from PAS and AMB make these varieties potential candidates to be exploited under climate change scenarios that include the joint action of rising CO_2_ and warming, since they can maintain fruit quality under those stressful environmental conditions; in contrast, TV shows a higher degree of phenotypic plasticity in response to increases in both air temperature and CO_2_ [9].

Rising CO_2_ can also induce the vegetative growth of grapevine, this effect being more marked when elevated CO_2_ interacts together with high air temperature and drought [10]. Although the effect of leaf-to-fruit ratio on the yield parameters, berry composition, and the balance between secondary and primary metabolites in fruits can strongly vary among grapevine varieties or cultivars [11,12], the regulation of the sink/source balance is considered as a powerful tool to adapt grape composition to technological objectives and increase wine quality [13]. Consequently, in a context of climate change that favors the vegetative growth of grapevine, a greater amount of vegetative residuals are obtained from summer pruning. These vegetative residuals are most times left in open fields and, to a lesser extent, used to feed cattle [14]. However, the known nutritional properties of grapevine leaves [15,16] move our attention toward using them for human nutrition. In fact, the cuisine of some Mediterranean areas (such as Egypt, Turkey, Greece, Lebanon, or Syria) includes leaves of grapevines for human consumption [17,18,19], as well as traditional medicines for the treatment of bleeding, inflammation, or diarrhea [20,21]. Moreover, Loizzo et al. [22] demonstrated the antiproliferative activity against human Caucasian breast adenocarcinoma of extracts prepared with leaves of Gaglioppo and Magliocco Dolce grapevine cultivars. Climate change can also affect the nutritional qualities and the cytotoxic potential of grapevine leaves. According to Torres et al. [23], the levels of minerals, proteins, sugars, and the antioxidant properties can be modified under warming conditions. The enhancement of air temperature can even increase the cytotoxic activity of grapevine leaves’ extracts against different cancer cell lines, thus opening the possibility of application of these vegetative residuals for biomedical uses [24]. Any of the mentioned options would allow the recycling of this underused plant waste, therefore promoting the circular economy.

Taking into account all these precedents, the objectives of the present study were (1) to evaluate the nutritional properties of the leaves of the abovementioned local Spanish grapevines when grown under environmental conditions that simulate climate change to understand their potential applications in gastronomy, as food supplements or as source of bioactive compounds, and in biomedicine, and (2) to assess to what extent the quality of the must obtained from those autochthonous Spanish grapevines is affected by increased CO_2_ concentration and warming, when acting alone or in interaction, to understand their potential as candidates to be cultivated under different climate change scenarios modulated by different geographic latitudes and altitudes.

## 2. Results

### 2.1. Nutritional Traits of Leaves

**Figure 1:**
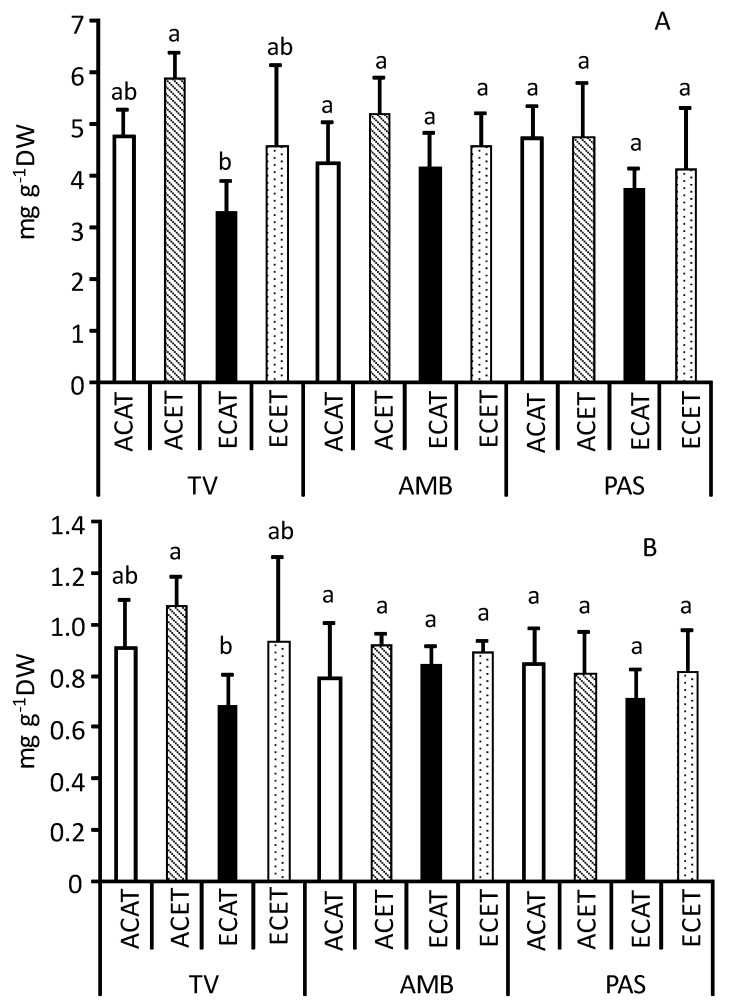
Concentrations of total chlorophylls (a + b) (mg g^−1^ DW) (**A**) and total carotenoids (mg g^−1^ DW) (**B**) in leaves of grapevine Tinto Velasco (TV), Ambrosina (AMB), and Pasera (PAS) grown at (1) ambient (~400 µmol mol^−^^1^) CO_2_ concentration (AC) and ambient temperature (AT) (ACAT); (2) AC and elevated (T + 4 °C, ET) temperature (ACET); (3) elevated CO_2_ (700 µmol mol^−^^1^, EC) and AT (ECAT), and (4) EC and ET (ECET). Bars represent means ± S.E. (n = 3–6). Within each graph and grapevine variety, bars topped by same letter indicate that values do not differ significantly (*p* > 0.05). DW = dry weight.

The concentrations of chlorophylls in leaves (ranging between 4.5 and 5 mg g^−1^ DW) were similar among grapevine varieties when cultivated at ambient CO_2_ and temperature conditions (ACAT) (as illustrated in Figure 1A). Increases in CO_2_ (ECAT), air temperature (ACET), or both (ECET) did not significantly affect the levels of chlorophylls in leaves of AMB and PAS. In contrast, the concentrations of chlorophylls in leaves of TV showed a downward trend when CO_2_ rose without interacting with increased temperature (ECAT) (as illustrated in Figure 1A), and this pattern was also found with carotenoids (as illustrated in Figure 1B).

**Figure 2:**
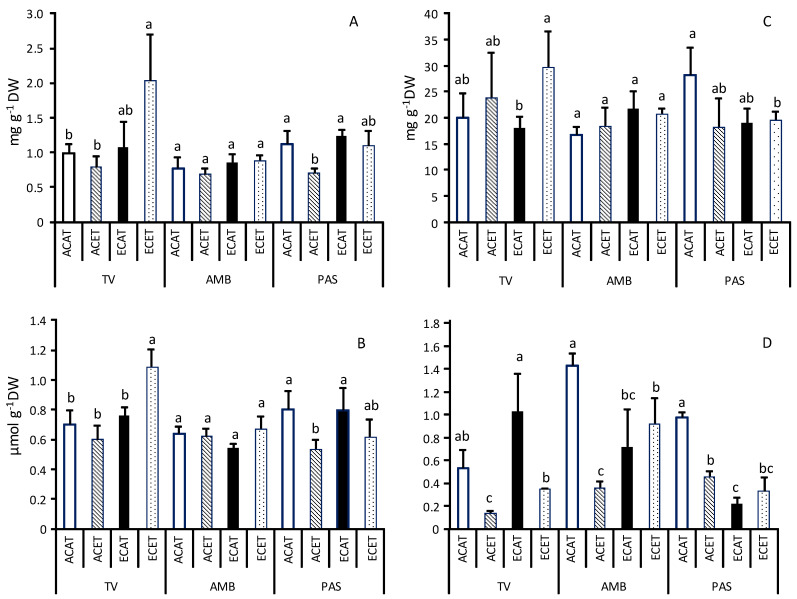
Concentrations of total soluble proteins (TSP) (mg g^−1^ DW) (**A**), proline (µmol g^−1^ DW) (**B**), total soluble sugars (TSS) (mg g^−1^ DW), (**C**) and starch (mg g^−1^ DW) (**D**) in leaves of grapevine Tinto Velasco (TV), Ambrosina (AMB), and Pasera (PAS) grown at (1) ambient (~400 µmol mol^−1^) CO_2_ concentration (AC) and ambient temperature (AT) (ACAT); (2) AC and elevated (T + 4 °C, ET) temperature (ACET); (3) elevated CO_2_ (700 µmol mol^−1^, EC) and AT (ECAT), and (4) EC and ET (ECET). Bars represent means ± S.E. (n = 3–6). Within each graph and grapevine variety, bars topped by same letter indicate that values do not differ significantly (*p* > 0.05). DW = dry weight.

Within each grapevine variety, the levels of total soluble proteins (TSP) (as illustrated in Figure 2A) and proline (as illustrated in Figure 2B) showed a parallel behavior. The three varieties had comparable concentrations of TSP (~1 mg g^−1^ DW, a little bit higher in PAS) and proline (between 0.7 and 0.8 µmol g^−1^ DW, being slightly higher in PAS) when cultivated at ACAT conditions. AMB showed high stability under environmental changing conditions. In contrast, leaves of TV accumulated higher levels of TSP (2 mg g^−1^ DW) and proline (1.1 µmol g^−1^ DW) when elevated CO_2_ interacted with increased air temperature (ECET), while the concentration of TSP and proline diminished in the leaves of PAS grown under elevated temperature acting alone (ACET).

The concentrations of sugars (TSS or starch) in leaves differed between the three varieties under ambient conditions (ACAT): although TV and AMB accumulated similar amount of total soluble sugars (TSS) (as illustrated in Figure 2C) (around 20 mg g^−1^ DW), the levels of starch (as illustrated in Figure 2D) were clearly higher in the leaves of AMB (1.5 mg g^−1^ DW) than in those of TV (around 0.6 mg g^−1^ DW). PAS was the variety with the greatest concentration of total sugars (TSS + starch) in leaves (around 30 mg g^−1^ DW) under ACAT conditions. The concentrations of starch (as illustrated in Figure 2D) were sensitive to changes in the environment, and the final result strongly differed among grapevine varieties. An increase in the air temperature not associated with rising CO_2_ caused significant reductions in the starch levels in the leaves of the three grapevine varieties. In AMB this negative effect of high air temperature on the starch concentrations was almost completely alleviated by the simultaneous application of elevated CO_2_ (ECET). In PAS the amount of starch in leaves was negatively affected by the elevated CO_2_ and increased temperature, acting independently (ECAT, ACET) or in combination (ECET). In contrast, elevated CO_2_ induced the accumulation of starch in leaves of TV under elevated temperatures (ECET vs. ACET).

**Figure 3:**
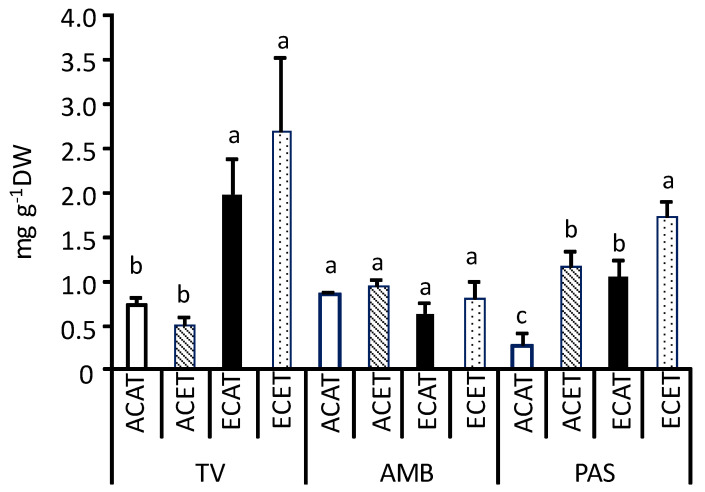
Concentrations of total soluble phenolic compounds (mg g^−1^ DW) in leaves of grapevine Tinto Velasco (TV), Ambrosina (AMB), and Pasera (PAS) grown at (1) ambient (~400 µmol mol^−1^) CO_2_ concentration (AC) and ambient temperature (AT) (ACAT); (2) AC and elevated (T + 4 °C, ET) temperature (ACET); (3) elevated CO_2_ (700 µmol mol^−1^, EC) and AT (ECAT), and (4) EC and ET (ECET). Bars represent means ± S.E. (n = 3–6). Within each graph and grapevine variety, bars topped by same letter indicate that values do not differ significantly (*p* > 0.05). DW = dry weight.

Under ambient conditions (ACAT), TV and AMB showed higher concentrations of total soluble phenolic compounds (0.8–0.9 mg g^−1^ DW) in leaves than PAS (0.3 mg g^−1^ DW) (as illustrated in Figure 3). The application of elevated CO_2_ and/or increased air temperature had not a significant effect on the levels of phenolics in leaves of AMB. In contrast, the concentrations of phenolics in leaves of TV increased between two or three times when cultivated under elevated CO_2_, either alone (ECAT) or simultaneously with increased air temperature (ECET). Similarly, the variety PAS was also sensitive to the environmental changes; in this case, elevated CO_2_ and high temperature induced the accumulation of phenolic compounds in leaves, both acting independently (ACET, ECAT) or in combination (ECET).

### 2.2. Quality Traits of Must

According to the ANOVA results, must characteristics in TV were significantly affected by the air temperature: pH, titratable acidity, soluble solids to titratable acidity ratio, and tonality index (as illustrated in Table 1). Some of these effects were reinforced when high temperature interacted with elevated CO_2_ (ECET): titratable acidity strongly decreased, the soluble solids to titratable acidity ratio increased the tonality index achieved the highest value. The CO_2_ factor induced a slight reduction of total soluble solids in TV. In contrast, must characteristics in PAS, and especially in AMB, remained mostly unchanged when plants underwent climate change conditions (as illustrated in Table 1). High temperature at ambient CO_2_ concentration (ACET) induced an increase in the pH of must of PAS, as well as in the tonality index in both AMB and PAS. In AMB, however, the increase in the tonality index was overshadowed by the interaction of temperature with elevated CO_2_ (ECET).

The phenolic composition of the TV must was strongly influenced by the environmental conditions (as illustrated in Table 2). The interaction between high temperature and elevated CO_2_ (ECET) significantly reduced extractable anthocyanins. However, the cellular extractability of anthocyanins (EA) increased under high temperature, both acting alone (ACET) and in combination with elevated CO_2_ (ECET). In AMB high temperature negatively affected TPI, total and extractable anthocyanins, the impact on anthocyanins being more pronounced under ambient CO_2_ (ACET). In contrast, high temperature did not influence the must phenolic composition in the PAS variety, but elevated CO_2_ reduced TPI regardless of temperature, and increased the EA at ambient temperature (ECAT).

## 3. Discussion

Antolín et al. [11] found that the fruit quality and must properties of local Spanish grapevine varieties were modified in different degrees when plants undergo high air temperature combined with elevated CO_2_ concentration; in fact, while berry and must traits significantly varied in some genotypes, in others these properties remain almost unaffected. In concrete, AMB and PAS belong to the first group, while TV belongs to the second one, which behaves quite similarly to the international variety Tempranillo. Antolín et al. [11], however, did not include information dealing with the effects of warm temperatures and elevated atmospheric CO_2_ on must characteristics when acting separately, nor did they assess the effects of those climatic factors on primary and secondary metabolites in leaves.

### 3.1. Nutritional Traits of Leaves

Defoliation is a cultural practice that improves the quality of grapes and wine. When applied in warm conditions, early leaf removal improved fruity and floral odor on Tempranillo [25]. In the present study, leaves were collected before flowering, thus simulating an early leaf removal performed in the field.

High temperature acting alone (ACET) did not affect the levels of soluble sugars (TSS) but did cause a marked decrease in the accumulation of starch in leaves of the three grapevine varieties (TV, AMB, and PAS). These results contrast with those obtained by Torres et al. [23] and Kizildeniz et al. [26] in cv. Red Tempranillo: the concentrations of TSS increased and those of starch did not change in the leaves when grown under warm temperatures. Such different behavior may be explained by both the genetic background of grapevine and the age of the leaves collected. Kizildeniz et al. [26] observed differences between Red and White Tempranillo in the response of leaf TSS to elevated temperature. Also, in the present experiment, the leaves were harvested during the early reproductive stages. In this period, leaves are an important source of carbohydrates for reserves restoration, developing vegetative organs, as well as for inflorescence and berry development [27], processes that may be hastened by warm temperatures, thus leading to a lower accumulation of starch in the leaves. In contrast, leaves in the studies of Torres et al. [23] and Kizildeniz et al. [26] were collected at fruit maturity when the amount of photosynthates destined to the bunch is becoming lower and the pool of starch reserves is already restored in the wood of most varieties [28]. In the present study, elevated CO_2_ did not produce an accumulation of carbohydrates in leaves, which was associated with limitations in increasing C sink strength in plants developed under high air CO_2_ [29,30]. Probably, the C sinks at the sampling stage (flowering stage) were strong enough to allow an adjustment in the C sink/source balance, thus maintaining and starch levels under elevated CO_2_ similar to those at ambient CO_2_.

Contrary to the common finding that the amount of proteins decreases in crops grown under elevated atmospheric CO_2_ concentrations [31], leaves of TV accumulated higher concentration of soluble proteins when cultivated under elevated CO_2_ interacting with high temperature (ECET), thus enhancing their nutritional value. The results agree with Martínez-Lüscher et al. [32], who reported a significant increase in total soluble proteins in the leaves of cv. Tempranillo grown under combined elevated CO_2_ and high temperature. Moreover, under these environmental conditions, the concentration of proline also increased, which possibly preserved the photosynthetic organs from oxidative stress by improving the antioxidant metabolism and reducing the lipid peroxidation and electrolyte leakage [33], thus protecting the biosynthesis of chlorophylls [34]. In fact, under ECET conditions, the concentrations of the photosynthetic pigments (chlorophylls and carotenoids) in leaves of TV were similar to those found under ambient CO_2_ and temperature (ACAT). From a nutritional point of view, chlorophylls and carotenoids may favor consumers’ health since they can delay the development of several chronic diseases [35] and prevent cardiovascular dysfunctions and cancer in human beings [36].

Elevated CO_2_ may increase the levels of antioxidants, including polyphenols, with a significant enhancement in the antioxidant capacity of different plant species [30]. The increase in the levels of total phenolic compounds observed in the leaves of TV and PAS under elevated CO_2_ (ECAT, ECET) and also in leaves of PAS grown at ambient CO_2_ and high temperature (ACET) may enhance the resistance of those grapevine varieties against diverse fungal diseases [37]. This circumstance could help to sustain culture systems preserving the environment since it may allow the reduction of the traditional Cu-based fungicides applied in vineyards [38]. Moreover, the well-known antioxidant properties of the natural phenolic compounds make these substances good candidates to be exploited as food supplements and functional ingredients in food and cosmetics [39]. Maia et al. [16] found that leaves of the grapevine variety Pinot Noir contain phenolic compounds and polyphenols with high antioxidant activity and proposed the exploitation of grapevine leaves as a source of bioactive compounds. This increased accumulation of total phenolic compounds observed in leaves of TV and PAS under elevated CO_2_ and/or high temperature, however, is not a universal pattern observed in plants subjected to these key factors of climate change. In fact, these environmental parameters did not affect the concentration of soluble phenolics in leaves of AMB. In addition, similar risings in the temperature and CO_2_ concentration in the air caused reductions in the amounts of total phenolics in the vegetative (stems and leaves) and reproductive (inflorescence) organs of the medicinal plant *Gynostemma pentaphyllum* [40].

In summary, data showed that the three local Spanish grapevines showed very different sensitivity to environmental parameters, with TV and AMB being the most and the least affected by air temperature and CO_2_ concentration, respectively. The interaction of elevated CO_2_ with high temperature (ECET) induced the accumulation of proteins and phenolic compounds in leaves of TV, thus enhancing their nutritional properties and possibly their resilience against fungal invasion. In PAS, the negative effect of high temperature (ACET) on the levels of proteins was alleviated when high temperature interacted with elevated CO_2_ (ECET).

### 3.2. Quality Traits of Must

The analysis of the main factors revealed that the significant reduction in the amount of total soluble solids in the must of TV exposed to the combination of warm temperatures and elevated CO_2_ (ECET) was mainly because of rising CO_2_. This result suggests that this local genotype has higher sensitivity to the increasing CO_2_ than some international varieties such as Riesling, Cabernet Sauvignon, and Tempranillo [12,41]. This apparent sensitivity of TV to rising CO_2_ is also supported by the significant interactions between temperature and CO_2_, which affected titratable acidity, tonality index, polyphenols, and anthocyanins. Concerning titratable acidity, the results of TV coincided with the general trend that high temperatures accelerate the decrease of grape acidity during ripening, mainly because of the faster depletion of malic acid [42]. However, berry acidity is also controlled in part by potassium, which partially neutralizes the negative charge of organic acids and may favor the formation of potassium tartrate precipitates. A high potassium level might explain the increase of must pH under ECET conditions in this variety. Elevated CO_2_ improved phenolic composition under ambient temperature (ECAT), which makes TV a promising candidate to be cultivated in latitudes or altitudes where the projected increase in atmospheric CO_2_ interacts less with high air temperatures. The increased accumulation of anthocyanins makes the must of TV interesting from a nutritional point of view due to the healthy properties of these secondary compounds [43]. Moreover, anthocyanins can protect grapes against the attack of some fungi, such as *Botrytis cinerea* [44].

Antolín et al. [11] suggested that the usually negative effect of warm temperatures on berry quality can be compensated for by the interaction of high temperature with elevated CO_2_. Results obtained with AMB corroborate this hypothesis because under ambient CO_2_ concentration, high temperature (ACET) significantly reduced the amount of anthocyanins in the must, and consequently, its color density, but it did not affect the levels of these compounds when combined with elevated CO_2_ (ECET). Similarly, in a recent study with five clones of Tempranillo, Arrizabalaga–Arriazu et al. [45] reported that the decrease in the anthocyanins to TSS ratio observed under elevated temperatures, with respect to ambient temperatures, disappeared when the high temperature treatment was combined with elevated CO_2_. Elevated CO_2_ at ambient temperatures (ECAT) induced the accumulation of polyphenols and did not influence the levels of anthocyanins. Taking all together, these results suggest that AMB could be an excellent candidate for exploitation in a future with increased air CO_2_ concentration both in warm and cool areas.

PAS behaved as the most stable of the three tested grapevines genotypes in terms of leaf characteristics, phenolic composition, and antioxidant activity of must when subjected to high temperature and/or elevated CO_2_, which indicates that this variety may be suitable to be cultivated in a broader geographical area under climate change scenarios without negatively affecting must quality.

In summary, warm climatic conditions were the most threatening scenario for maintaining the quality of the must in the three assessed local Spanish grapevines. Elevated CO_2_ exerted a beneficial effect when acting alone and by alleviating the negative effects of high temperatures. PAS behaved as the most stable genotype under different environmental scenarios, making it the most versatile candidate for cultivation in areas affected by climate change.

## 4. Materials and Methods

### 4.1. Plant Material and Growth Conditions

The selection of the grapevine varieties included in this study was based on previous data obtained in the Estación de Viticultura y Enología de Navarra (EVENA). This plant material fulfilled the following premises: suitable sanitary conditions (including virus-free), collected from vines with distinctive morphological and ampelographic characters [46]. Vines referenced and marked in the field were genetically identified by molecular markers and properly preserved. Molecular profiles with 8 microsatellites markers of the three varieties are presented in Table 3.

The molecular identification was carried out using 8 microsatellites markers (VMC4F3-1 VVIN16, VVIV37, VVIV67, VVMD27, VVIP31, VVS2, and ZAG79) in the frame of the Project RF2012-00027-C05- 02 at the Estación Experimental de Aula Dei-CSIC. Briefly, DNA from fresh leaves was extracted with Qiagen Dneasy Plant Mini kit (Qiagen, Hilden, Germany) according to the manufacturer’s instructions. For amplification, 5 ng of DNA template containing 1X PCR Master Mix of QIAGEN kit multiplex PCR (Qiagen, Hilden, Germany) in two multiplex PCRs (A and B) were performed in a 2700 thermal cycler (Applied Biosystems^®^), and fragments were analyzed on the ABI PRISM DNA sequencer (3130XL; Applied Biosystems, Foster City, CA, USA). Allelic sizes were assigned using the GeneMapper v4.1 software (Applied Biosystems, Foster City, CA, USA), and then profiles were compared with that of the El Encin germplasm bank database to identify the genotype number (IMIDRA, Madrid, Spain).

The three genotypes were grown in an experimental vineyard located in Olite (Navarra, Spain) (latitude: 42°29′15″ N; longitude: 1°39′45″ W; altitude: 388 mamsl) and displayed contrasting features for the bunch and berry mass (as illustrated in Table 4). Dormant cuttings of each variety were collected after the winter pruning, and those that were 400–500 mm in length were induced for fruit-bearing according to the steps originally outlined by [47], and then modified by [48]. Rooting was induced by immersing the cuttings in a solution of indole-3-butyric acid (400 mg L^−1^) and placing them in a warm bed (27 °C) in a cold room (4 °C) for 30 days. Once rooting was successful, the own-rooted cuttings were planted in 0.8 L plastic pots containing perlite and peat (1:1 *v*:*v*), and then transferred to a greenhouse. Initial growth conditions were 25/15 °C and 50/90% relative humidity (day/night) regime and natural daylight (photosynthetic photon flux density, PPFD, was on average 850 μmol m^−2^ s^−1^ at midday) supplemented with high-pressure sodium lamps (OSRAM, Augsburg, Germany) to extend the photoperiod up to 15 h. Under these conditions, bud-break occurred after 7–8 days, and from this moment the growth was controlled until flowering, leaving only one inflorescence and 4 leaves per plant. While flowering in AMB, TV and PAS started, respectively, on 9, 14, and 17 May 2019, fruit set occurred on 19, 20, and 25 May 2019.

### 4.2. Experimental Design

After fruit set (Eichhorn and Lorenz (E–L) growth stage 27) [49], which took place about one month after bud-break, plants were transplanted to 13 L plastic pots containing perlite and peat (1:1 *v*:*v*). Afterwards, plants were transferred to four temperature gradient greenhouses (TGGs) located at the University of Navarra (Pamplona, Spain) (latitude: 42°49′00″ N; longitude: 1°39′00″ W; altitude: 450 mamsl) for the application of the CO_2_ and temperature treatments. Two TGGs were used for the ambient CO_2_ treatment (ACO_2_), and two TGGs were used for the elevated CO_2_ treatment (ECO_2_). In the two ambient CO_2_ (ACO_2_) greenhouses, no CO_2_ was added and [CO_2_] in the atmosphere was ~400 µmol mol^−1^. In the other two elevated CO_2_ (ECO_2_) greenhouses, [CO_2_] was fixed at ~700 μmol mol^−1^ by injecting pure CO_2_ (purity up to 99.99%) from cylinder-gases (34 L of CO_2_ per cylinder) at the two inlet fans during the light hours. The CO_2_ was provided by Carburos Metálicos S.A. (Air Products Group, Cornellà de Llobregat, Barcelona). The [CO_2_] was continuously monitored using a Guardian Plus gas monitor (Edinburgh Instruments Ltd., Livingston, UK). The monitor’s signal was fed into a proportional integrative differential controller that regulated the opening time (within a 10 s cycle) of a solenoid valve which injected CO_2_ into both inlet fans.

The four TGGs have a modular design with three temperature modules (3.04 m long each). Within each TGG a temperature gradient is created (from module 1 of ambient temperature to module 3 of ambient temperature +4 °C) by circulating air to maintain the difference of 4 °C between modules (for more details see [50]). Module 2 had no plants because is a module of transition. Inside the greenhouses, the pots were placed in holes made in the soil to simulate natural temperature fluctuations, thus keeping the temperature differences between shoots and roots found under field conditions [51].

Plants of the three varieties were randomly distributed into the TGGs, and four climate conditions were set up: (1) ambient CO_2_ (around 400 µmol mol^−1^) and ambient temperature (T) (ACAT); (2) ambient CO_2_ and elevated temperature (T + 4 °C) (ACET); (3) elevated CO_2_ (700 µmol mol^−1^) and ambient temperature (ECAT), and (4) elevated CO_2_ and elevated temperature (ECET). The vegetative growth of plants was controlled by manual summer pruning to maintain an adequate leaf area to fruit mass ratio for berry ripening (ca. 14 leaves per plant) [52]. The irrigation (both at the pretreatment greenhouse and at the TGGs) was performed using a nutritive solution according to the grapevine requirements [48] alternated with deionized water to prevent both clogging of drippers in the irrigation system and salinization of the substrate. Plants were watered twice per day and irrigation doses were adjusted according to the needs of the plant throughout its fruit development. Quartz stones were used on the surface of the pots to avoid evaporation and consequently, excessive loss of water from the substrate. There were 3–6 biological replicates for each combination of CO_2_, temperature, and variety. Plants remained in the TGGs until berries reached commercial maturity (E–L 38 stage).

### 4.3. Harvest of Leaves and Determinations of Metabolites

Plant material used for the determination of pigments, primary and secondary metabolites were fully expanded and functional leaves which completed their whole development under the different environmental conditions previously explained. Leaves collected around two months before maturity (and harvest) of fruits were those obtained during summer pruning process performed to maintain the ratio between leaf area and fruit mass.

To express the results on a dry basis (DW), for every environmental treatment leaf, samples of known FW were oven dried at 70 °C until weight was constant. Then the ratio of fresh to dry weight was calculated for each sample, and the average value was applied to the concentrations of TPS, TSS, proline, starch, and phenolic compounds initially expressed by FW.

Total chlorophylls (a + b) and total carotenoids were extracted according to Sèstak et al. [53] by immersing samples of fresh leaves (1.3 cm^2^) in 5 mL of 96% ethanol at 80 °C for 10 min. The absorbance of extracts was measured at 470, 649, 665, and 750 nm. Estimation of total chlorophylls (a + b) and total carotenoids was performed using the extinction coefficients and equations described by Lichtenthaler [54]. Results were expressed as mg of chlorophylls or carotenoids per g of leaf DW.

Determination of total soluble proteins (TSP), total soluble sugars (TSS), proline, and starch was performed on 0.5 g of fresh leaves (0.5 g FW) which were ground in an ice-cold mortar and pestle containing potassium phosphate buffer (50 mM, pH 7.0). The homogenates were filtered through four layers of cheese cloth and centrifuged at 5000 rpm at 4 °C for 15 min. The supernatant was collected and stored at 4 °C for TSP, TSS, and proline determinations. The pellet was used to determine starch after iodine reaction [55]. TSP were analyzed with the protein dye-binding method [56] and TSS with the anthrone reagent [57] using bovine serum albumin (BSA) and glucose as standards, respectively. Proline was colorimetrically estimated (515 nm) by the ninhydrine reaction [58]. Results were expressed as mg of TPS, TSS, and starch per g of leaf dry weight (DW) and as µmoL proline per g of leaf DW. 

Total phenolic compounds were extracted according to Chapuis–Lardy et al. [59] with some modifications. Samples of fresh leaves (0.2 g FW) were pulverized in liquid nitrogen, mixed with 20 mL of 80% methanol, and homogenized at room temperature for 1 min. After filtration, 0.5 mL of each sample were mixed with 10 mL of distilled water. Total phenolic content was determined from aqueous solutions by spectrophotometric analysis at 760 nm with Folin–Ciocalteau reagent [60] Results were expressed as mg of gallic acid per g of leaf DW.

### 4.4. Harvest of Berries and Quality Determinations

Fruit maturity was established by sampling periodically two or three berries to measure the total soluble solids (°Brix) and titratable acidity in the must. Following expert advice from the EVENA, every plant was harvested when the ratio sugars (°Brix) to acidity ranged between 4 and 6. Berries were frozen at −20 °C for further analysis. 

A subsample of 20 berries was crushed and filtered through gauze, and then extracts were centrifuged at 4100× *g* at 4 °C for 10 min. The supernatant was used for the following determinations: total soluble solids measured with a temperature-compensating refractometer (Zuzi model 315; Auxilab, Beriáin, Spain) and expressed as °Brix; must pH measured with a pH meter (Crison Instruments, Barcelona, Spain) standardized to pH 7.0 and 4.0; titratable acidity measured by titration with NaOH according to International Organization of Vine and Wine methods [61]. Another 20-berry subsample per plant was taken for the analysis of anthocyanins, total phenols, and chromatic properties. Total and extractable anthocyanins were determined according to the procedure described by Saint–Cricq et al. [62]. Two samples of the nonfiltered, crushed grape homogenate were macerated for 4 h at pH 1 (hydrogen chloride) and pH 3.2 (tartaric acid), respectively. Then, the macerated samples were centrifuged at 4,100× *g* at 4 °C for 10 min. Total and extractable anthocyanins were determined in both supernatants (macerated at pH 1 and pH 3.2) following the protocol of Ribéreau–Gayon and Stonestreet [63] by reading absorbance at 520 nm. Both data were used to calculate the cellular extractability (EA) of anthocyanins [64]. The seed maturity (SM) index was calculated by the Glories method [64]. Total polyphenol index (TPI) was calculated by the absorbance reading at 280 nm in the supernatant obtained after maceration at pH 3.2 [65]. Color density was calculated by adding the absorbance readings at 420, 520, and 620 nm, whereas tonality index was determined as the ratio of absorbance readings at 420 and 520 nm of the samples extracted at pH 3.2 [66]. 

Total antioxidant capacity was evaluated on the same must samples used for berry quality determinations by using the free-radical scavenging activity (α, α-diphenyl-β-picrylhydrazyl, DPPH) assay [67]. The free radical scavenging activity, using the free radical DPPH, was evaluated by measuring the variation in absorbance at 515 nm at 25 °C. The reaction was started by adding 25 µL of the corresponding sample to the cuvette containing 80 mM (methanol solution) (975 µL) of the free radical (DPPH *). The final volume of the assay was 1 mL. The reaction was followed over 15 min with a spectrophotometer. The calibration curve was made using gallic acid as a standard, and results were expressed as mg gallic acid per mL of must.

### 4.5. Statistical Analyses

Statistical analyses were carried out using statistical software the Statistical Package for the Social Sciences (SPSS) (SPSS Inc., Chicago, IL, USA) version 21.0 for Windows. After establishing the normal distribution of the residuals with the Kolmogorov–Smirnov normality test due to the small sample size (n = 3–6) and the homogeneity of variance with the Levene test, data within each clone were subjected to a two-way analysis of variance (ANOVA) with or without Welch correction, taking into account whether the requirement of the homogeneity of variances was fulfilled or not. The test allowed assessing the main effect of the factors’ concentration of CO_2_ in the atmosphere, (CO_2_: ACO_2_ and ECO_2_) and temperature (T: T and T + 4 °C), and the interaction between them. Means ± standard errors (SE) were calculated, and when the F ratio was significant (*p* ≤ 0.05), a Duncan test was applied.

## 5. Conclusions

TV was the most sensitive grapevine genotype to the environmental conditions since different air temperatures and CO_2_ concentrations in the air, acting separately or in combination, affected the metabolism of both vegetative (leaves) and reproductive (fruits) organs. However, the environmental conditions that most improved the nutritional properties of leaves were not the most favorable for the must quality, thus limiting the reuse in optimal conditions of early-removed leaves as a source of nutraceutical compounds. Our results suggest that under the elevated air CO_2_ conditions projected by the end of this century, TV may be a candidate to be cultivated in not very warm areas (higher altitudes or colder latitudes). Under such conditions, leaves and fruits of TV may also have enhanced tolerance against fungal diseases thanks to the increased accumulation of phenolic compounds. In AMB, the warm temperature had a negative effect on fruit quality when acting alone, but this effect was compensated for by elevated CO_2_. In this grapevine variety, the accumulation of metabolites in the vegetative organs was little influenced by the environmental conditions, thus maintaining their nutritional properties under all climate change scenarios simulated in our study. By contrast, the high stability shown by PAS in terms of fruit quality when exposed to different temperatures and concentrations of CO_2_ in the air was not observed in the leaves. In fact, under climate change scenarios leaves accumulated greater amounts of phenolic compounds. Therefore, the most adequate environmental conditions for exploiting AMB and PAS as wine grapevines would be also appropriate for maintaining (AMB) or even improving (PAS) the nutritional properties of leaves, making these vegetative residuals an interesting recycled product as a source of bioactive nutraceutical substances. Further studies will be performed to compare the behavior of these minority grapevine varieties with that of other widely cultivated red varieties (Tempranillo, Garnacha, Merlot, and Cabernet Sauvignon) when subjected to similar environmental conditions.

## Figures and Tables

**Table 1:** Must characteristics in three Spanish autochthonous grapevine varieties cultivated at (1) ambient (~400 µmol mol^−^^1^) CO_2_ concentration (AC) and ambient temperature (AT) (ACAT); (2) AC and elevated (T + 4 °C, ET) temperature (ACET); (3) elevated CO_2_ (700 µmol mol^−^^1^, EC) and AT (ECAT), and (4) EC and ET (ECET). Values are means (n = 3–6). Within each parameter and grapevine variety, values followed by same letter or with absence of letters do not differ significantly (*p* ≥ 0.05). Significance of analysis of variance (ANOVA): *** *p* ≤ 0.001; ** *p* ≤ 0.01; * *p* ≤ 0.05; ns, not significant (*p* ≥ 0.05). T = temperature factor.

	Treatments				ANOVA		
	ACAT	ACET	ECAT	ECET	CO_2_	T	CO_2_ × T
Tinto Velasco (TV)							
Total soluble solids (°Brix)	20.3 a	18.1 ab	17.0 b	16.9 b	*	ns	ns
Must pH	3.95 b	4.03 ab	3.84 b	4.26 a	ns	**	ns
Titratable acidity (g L^−^^1^)	4.85 b	3.55 ab	3.05 a	2.69 c	ns	*	***
Soluble solids/Titratable acidity	4.30 b	5.20 ab	5.66 a	6.28 a	ns	*	***
Color density	2.63 a	2.57 a	2.53 ab	1.95 b	ns	ns	ns
Tonality index	0.77 c	0.92 b	0.75 c	1.28 a	***	***	***
Ambrosina (AMB)							
Total soluble solids (°Brix)	20.9	20.4	20.3	21.4	ns	ns	ns
Must pH	3.93	3.76	3.82	4.03	ns	ns	ns
Titratable acidity (g L^−^^1^)	4.64	4.88	5.38	4.15	ns	ns	ns
Soluble solids/Titratable acidity	4.73	4.37	4.06	5.28	ns	ns	ns
Color density	2.22 ab	1.59 b	2.44 a	2.40 a	ns	ns	ns
Tonality index	0.78 b	1.14 a	0.74 b	0.84 b	*	**	*
Pasera (PAS)							
Total soluble solids (°Brix)	21.0 a	21.7 a	22.9 a	22.2 a	ns	ns	ns
Must pH	3.91 b	4.24 a	4.00 ab	4.21 a	ns	**	ns
Titratable acidity (g L^−^^1^)	3.94 a	4.18 a	4.88 a	4.68 a	ns	ns	ns
Soluble solids/Titratable acidity	5.46 a	5.36 a	4.76 a	5.09 a	ns	ns	ns
Color density	2.46 a	2.24 a	2.18 a	2.34 a	ns	ns	ns
Tonality index	0.77 b	0.90 a	0.80 ab	0.90 a	ns	**	ns

**Table 2:** Phenolic composition and antioxidant capacity of must from three Spanish autochthonous grapevine varieties cultivated at (1) ambient (~400 µmol mol^−^^1^) CO_2_ concentration (AC) and ambient temperature (AT) (ACAT); (2) AC and elevated (T + 4 °C, ET) temperature (ACET); (3) elevated CO_2_ (700 µmol mol^−^^1^, EC) and AT (ECAT), and (4) EC and ET (ECET). Values are means (n = 3–6). Within each parameter and grapevine variety values followed by same letter or with absence of letters do not differ significantly (*p* ≥ 0.05). Significance of analysis of variance (ANOVA): *** *p* ≤ 0.001; ** *p* ≤ 0.01; * *p* ≤ 0.05; ns, not significant (*p* ≥ 0.05). T = temperature factor. TPI = Total polyphenol index; AU = absorbance units; EA = Cellular extractability of anthocyanins; SM = Seed maturity.

	Treatments				ANOVA		
	ACAT	ACET	ECAT	ECET	CO_2_	T	CO_2_ × T
Tinto Velasco (TV)							
TPI (AU)	12.3 b	12.4 b	22.5 a	8.6 b	ns	**	***
Total anthocyanins (mg L^−1^)	210 b	350.8 a	352.8 a	148.3 b	ns	ns	***
Extractable anthocyanins (mg L^−1^)	157.2 ab	126.3 b	182 a	58.6 c	ns	***	*
EA (%)	29.4 b	63.6 a	49.0 a	56.8 a	ns	***	ns
SM (%)	54.0 b	57.9 ab	66.4 ab	71.4 a	*	ns	ns
Total antioxidant capacity (mg L^−1^)	18.0 a	119.8 a	19.7 a	20.0 a	ns	ns	ns
Ambrosina (AMB)							
TPI (AU)	23.1 ab	20.7 b	32.4 a	16.2 b	ns	*	ns
Total anthocyanins (mg L^−1^)	200.0 a	98.6 c	153.8 ab	133.0 bc	ns	***	ns
Extractable anthocyanins (mg L^−1^)	157.4 a	61.3 c	152.8 ab	104.7 bc	ns	***	ns
EA (%)	22.9 a	24.8 a	8.8 bc	17.3 ab	**	ns	ns
SM (%)	65.1	76.8	75.7	73.9	ns	ns	ns
Total antioxidant capacity (mg L^−1^)	17.4 b	21.9 ab	20.0 ab	22.8 a	ns	*	ns
Pasera (PAS)							
TPI (AU)	32.4 a	29.3 ab	21.6 b	20.3 b	*	ns	ns
Total anthocyanins (mg L^−1^)	155.2	155.3	156.1	152.7	ns	ns	ns
Extractable anthocyanins (mg L^−1^)	152.4	139.1	131.6	134.4	ns	ns	ns
EA (%)	11.4 b	18.8 b	30.4 a	16.0 b	*	ns	ns
SM (%)	77.5	80.3	66.2	67.2	ns	ns	ns
Total antioxidant capacity (mg L^−1^)	21.5	20.3	22.6	19.9	ns	ns	ns

**Table 3:** Code, passport number, variety name, origin, genotype, and molecular profiles (allele sizes in base pairs) of varieties identified with 8 microsatellite markers.

Code	Passport	Variety/Local Name	Origin	Genotype	VMC4F3-1		VVIN16		VVIV37		VVIV67		VVMD27		VVIP31		VVS2		ZAG79	
T73	CS0073	Tinto Velasco/unknown	Los Arcos (Navarra)	GEN 0062	187	206	151	151	158	158	358	375	179	185	184	190	131	131	237	251
T46	CS0046	Ambrosina/Tinta	Los Arcos (Navarra)	GEN 0906	167	187	151	153	163	165	362	372	179	194	184	190	131	131	251	251
T85	CS0085	Parrel/Pasera	Ablitas (Navarra)	GEN 0101	179	187	153	159	161	171	364	372	179	194	176	180	141	151	251	257

**Table 4:** Agronomic characteristics of Spanish autochthonous grapevine (*Vitis vinifera* L.) varieties used in present study. Data were provided by Estación de Viticultura y Enología de Navarra (Olite, Spain) and obtained from plants cultivated in vineyard in 2019. FW = fresh weight.

Variety	Code	Reproductive Cycle	Bunch Mass (g FW Bunch^−^^1^)	Berry Mass (g FW Berry^−^^1^)
Tinto Velasco (TV)	T73	Medium	153	2.01
Ambrosina (AMB)	T46	Long	205	1.38
Pasera (PAS)	T85	Long	331	2.02

## Data Availability

All data, tables and figures in this manuscript are original. Data files of the present study were deposited at the Department of Environmental Biology, School of Sciences, University of Navarra.
